# Histologic factors associated with nintedanib efficacy in patients with idiopathic pulmonary fibrosis

**DOI:** 10.1371/journal.pone.0245147

**Published:** 2021-01-07

**Authors:** Masahiro Nemoto, Yoshiaki Zaizen, Kensuke Kataoka, Kishio Kuroda, Kazuhiro Tabata, Andrey Bychkov, Hiromitsu Sumikawa, Takeshi Johkoh, Masahiro Aoshima, Yasuhiro Kondoh, Junya Fukuoka

**Affiliations:** 1 Department of Pulmonary Medicine, Kameda Medical Center, Kamogawa, Japan; 2 Department of Immunology, Graduate School of Medicine, Chiba University, Chiba, Japan; 3 Department of Pathology, Nagasaki University Graduate School of Biomedical Sciences, Nagasaki, Japan; 4 Department of Respiratory Medicine and Allergy, Tosei General Hospital, Seto, Japan; 5 Department of Pathology, Kameda Medical Center, Kamogawa, Japan; 6 Department of Diagnostic Radiology, Sakai City Medical Center, Sakai, Japan; 7 Department of Radiology, Kinki Central Hospital of Mutual Aid Association of Public School Teachers, Itami, Japan; Rutgers Biomedical and Health Sciences, UNITED STATES

## Abstract

**Background:**

Histopathologic factors predictive of nintedanib efficacy in idiopathic pulmonary fibrosis have not been studied. We aimed to describe the characteristics, focusing on histopathology, of idiopathic pulmonary fibrosis patients who did and did not respond to nintedanib.

**Methods:**

This study retrospectively examined the clinicoradiopathologic features of 40 consecutive patients with surgical lung biopsy-confirmed idiopathic pulmonary fibrosis treated with nintedanib. Additionally, we compared the histopathologic scoring of 21 microscopic features between patients with functional or radiological progression and those with non-progression during 12 months of treatment.

**Results:**

The histopathologic evaluation showed edematous changes in the interlobular septum as the only histologic finding observed more frequently in patients with both functional and radiological progression than in those without (58% vs. 14%, P = 0.007 and 50% vs. 0%, P = 0.003, respectively). Regarding per-year change, patients with edematous changes in the interlobular septum showed greater progression in median changes in spared area (-12%, interquartile range: [-25%–-5%], vs. -3% [-7%–0%], P = 0.004) and reticular shadow (7% [3%–13%], vs. 0% [0%–5%], P = 0.041) on computed tomography. Functional and radiological progression-free survival were shorter in patients with edematous changes in the interlobular septum than in those without (6.6 months, 95% confidence interval: [5.9–25.3], vs. event <50%, [12.1–Not available], P = 0.0009, and 6.1 months, [5.2–6.6] vs. 14.5 months [7.8–not available], P<0.0001).

**Conclusions:**

Edematous changes in the interlobular septum may indicate poor nintedanib efficacy in idiopathic pulmonary fibrosis. Further studies are needed to validate these findings and address the mechanism behind ECIS.

## Introduction

Predictive biomarkers of nintedanib efficacy in patients with idiopathic pulmonary fibrosis (IPF) have not been addressed previously but are urgently needed for optimal clinical outcomes. As a study of serum markers is already underway [1], a histopathological investigation is of equal importance. IPF is a severe progressive fibrotic disease of unknown cause that results in intractable dyspnoea upon exertion. The disease has a reported median overall survival period of 30 months—a time comparable to that of stage III non-small cell lung cancer [2, 3].

Alveolar epithelium and fibroblasts are thought to be key players in the pathogenesis of IPF because recurrent epithelial injury (EI) leads to aberrant repair of the injured alveoli and deposition of interstitial fibrous tissue by myofibroblasts [4]. The 2015 updates to the American Thoracic Society, European Respiratory Society, Japanese Respiratory Society, and Latin American Thoracic Association IPF treatment guidelines list antifibrotic agents, such as nintedanib and pirfenidone, as conditional recommendations [5]. Nintedanib is a triple tyrosine kinase inhibitor that targets vascular endothelial, fibroblast, and platelet-derived growth factor receptors [4]. Results from a phase 3 clinical trial [6] and a meta-analysis [7] indicated that nintedanib lessens the decline in forced vital capacity (FVC); prospective cohort studies [7, 8] also demonstrated that nintedanib therapy is associated with a lower rate of acute exacerbation and a lower hazard ratio (HR) for all-cause deaths. The development of effective treatments has stressed the need for timely and accurate diagnosis because of the heterogeneity of IPF and the ability of other interstitial pneumonias to mimic IPF [4]. Thus, multidisciplinary discussions are important for accurate diagnosis and better patient prognosis [9].

Another clinical problem associated with IPF is its unpredictable response to antifibrotic treatment. Although antifibrotics showed a 50% reduction of FVC decline in 1 year, compared with the placebo group in a phase 3 trial and recent network meta-analysis, the individual response to antifibrotics is unpredictable [6, 10]. Predictive biomarkers for antifibrotic treatment should be elucidated to establish better treatment strategies. Although biomarkers of disease stability and pathological prognostic factors have been identified [2, 11–14], few studies have investigated predictive factors of IPF treatment outcomes [15]; even fewer have examined pathological specimens.

Thus, the aim of this study was to describe the histopathologic characteristics of patients with IPF who did and did not respond to nintedanib treatment.

## Materials and methods

### Study patients

Consecutive patients with IPF, who started nintedanib (200 or 300 mg/day) treatment by January 2017, were retrospectively identified from electronic medical records at two tertiary care hospitals. IPF was diagnosed by a multidisciplinary team using multimodal information, including chest computed tomography (CT) scans and surgical lung biopsy (SLB) findings. The exclusion criteria were: (1) loss to follow-up within 6 months from starting nintedanib treatment; (2) concomitant medication (e.g., systemic glucocorticosteroids, immunosuppressants, or pirfenidone) use when starting nintedanib treatment; (3) missing data within 6 months prior to starting nintedanib treatment and at 12 months after the start of nintedanib treatment, except for cases of death or acute exacerbation; and (4) unresectable lung cancer.

### Study design

This was a two-centre, retrospective, cohort study of patients with IPF in Japan that assessed the characteristics of patients with IPF who did and did not demonstrate functional or radiological progression. Functional progression was defined as the presence of indicators based on the following composite factors after 12 months of nintedanib treatment: 1) all-cause death; 2) acute exacerbation, reported by pulmonary physicians; 3) relative FVC decline of ≥10% [16]. Radiological progression was defined similarly: 1) all-cause death; 2) acute exacerbation; and 3) relative change in the extent of reticular opacity or honeycombing (HC), or reduction in the extent of normal lung area on chest CT scans of ≥10%. Functional and radiological progression included all-cause death and acute exacerbation to avoid competing-risk bias and was defined as fulfilment of at least one of the composite progression criteria; failure to fulfil any of the criteria was classified as non-progression. The study design and reporting adhered to the Strengthening the Reporting of Observational Studies in Epidemiology Statement [17]. The institutional review boards of both hospitals (Kameda Medical Center, Kamogawa, Japan, and Tosei General Hospital, Seto, Japan) approved the study and waived the need for written informed consent from the study patients. (Approval no.: 17–089 and 674, respectively).

### Data sources and measurements

Baseline clinical, pulmonary function test (PFT), and chest CT data were obtained from the electronic medical records of both hospitals. Clinical data included patient age, sex, smoking status, and comorbid diseases. PFT and chest CT data were obtained before the start of nintedanib treatment (within 3 months), as well as at a variable point of 6 to 12 months after starting nintedanib treatment.

Chest CT scans were obtained with 1- or 2-mm collimation at 1- or 10-mm intervals and fitted under a high spatial frequency algorithm. Two blinded board-certificated chest radiologists, with 30 and 16 years of experience reviewed the chest CT data. The images were independently evaluated to score the extent of reticular opacity, HC, traction bronchial-bronchiolectasis, ground-glass opacity, airspace consolidation, cyst formation, emphysema (Em), and spared lung area in gradations of 5%, as previously described by Fujisawa et al. [18]. Consensus was established when the numerical difference between the evaluations differed by ≥10%; the inter-rater variability was assessed.

### Histological evaluation

Whole-section SLB specimens were stained using haematoxylin and eosin and Elastica van Gieson or Elastica–Masson staining. All evaluations were performed using digitized images by slide scanners at 20× magnification (Aperio CS, Sausalito, CA, USA or VS100, Olympus, Tokyo, Japan). The SLB images were independently evaluated by four board-certificated pulmonary pathologists, with 9–24 years of experience, blinded to the clinical data. The histopathological findings were rated from 0 to 3 points (0, absent; 1, weak; 2, moderate; and 3, strong) and further dichotomized as negative (0–1 points) or positive (2–3 points). Consensus was established when the positive or negative judgements were discrepant among the reviewers; the inter-rater variability was assessed. The histopathological findings of interest (total– 21 features) were: dense fibrosis (DF), cellular interstitial pneumonia, marked organizing pneumonia, fibroblastic focus (FF), destroyed architecture (DA), HC, smooth muscle hyperplasia, elastosis, lymphoid follicles, cellular/fibrinous pleuritis, plasma cell infiltration, constrictive bronchiolitis, vascular intimal thickness, loose granuloma, interstitial giant cells, peribronchiolar metaplasia, airway-centred changes, EI, Em, alveolar exudate, and edematous changes in the interlobular septum (ECIS). All pathologists had a group session to standardize the morphologic features prior to their review of the study specimens.

### Statistical analysis

Baseline clinical characteristics and histological and radiological data were expressed as descriptive statistics. The study outcome was analysed relative to each variable. Progression-free survival (PFS) was calculated from the first day of nintedanib treatment until progression status assessments at both the 6- and 12-month time points. The χ^2^ test was performed to analyse categorical variables and the Mann–Whitney *U* test was performed to analyse continuous variables. Logistic regression analysis was performed as multivariate analysis. Statistical significance was indicated by a P-value <0.05. All analyses were performed using the R statistical software package (version 3.5.2, R Foundation for Statistical Computing, Vienna, Austria) with the add-on EZR package (version 1.36, Saitama Medical Center, Jichi Medical University, Saitama, Japan) [19].

## Results

### Study patients

**Fig 1:**
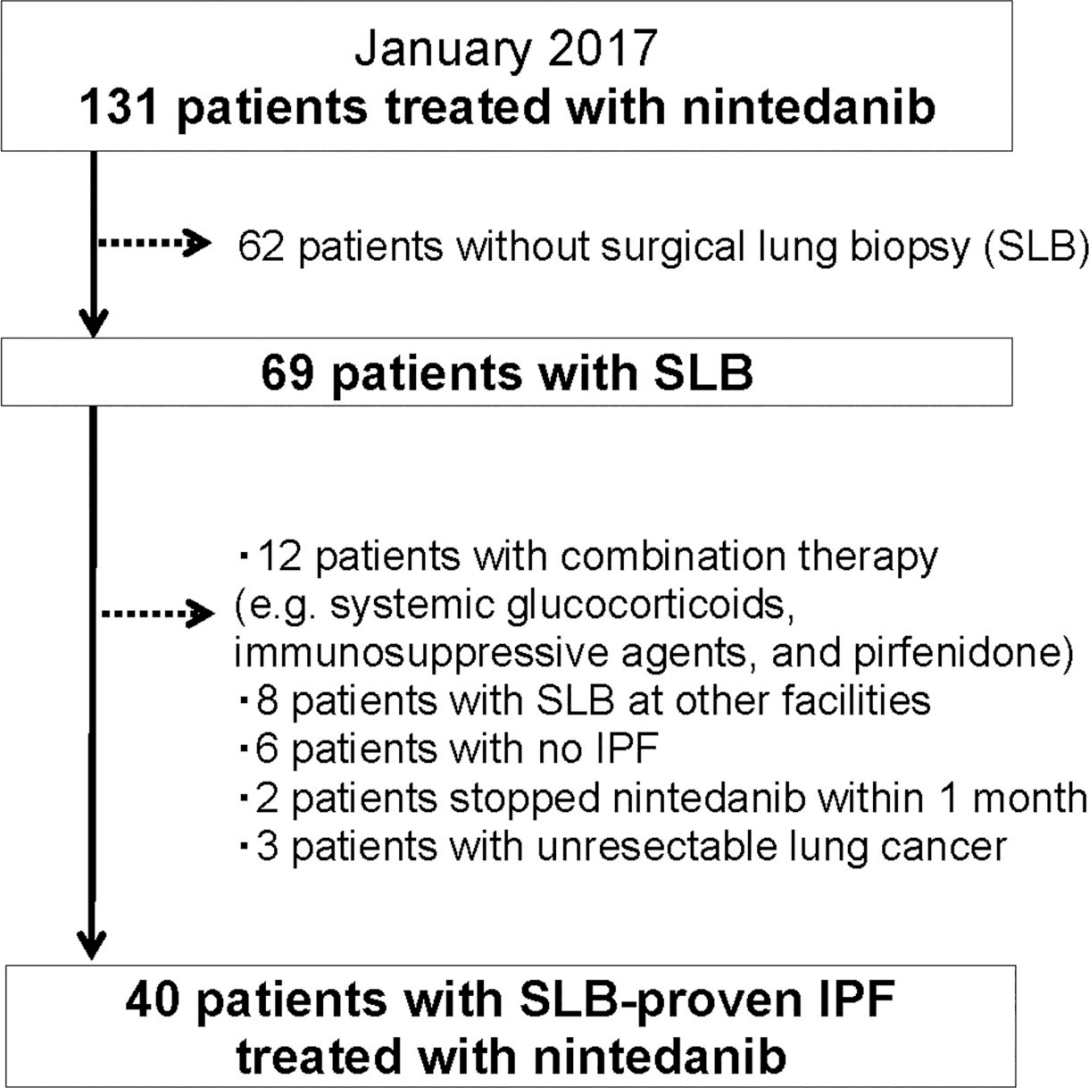
Patient selection flow chart.

A total of 131 patients with IPF were undergoing treatment with nintedanib at the study hospitals by January 2017. After excluding 62 patients without SLBs, 12 receiving combination therapy with an immunosuppressant, eight with SLBs performed at other hospitals, six with misdiagnoses, two who discontinued nintedanib treatment within 1 month, and three with unresectable lung cancer, 40 patients ultimately met the study criteria (Fig 1, S1 Dataset). All 40 SLBs were performed in multiple lobes including the lower lobe. The baseline characteristics of the 40 patients are presented in Table 1.

**Table 1:** Patients characteristics.

Characteristics	Study cohort (n = 40)
Age, median, years (IQR)	66 (64, 70)
Men, n (%)	32 (80)
Smoking status, n (%)	
Non-smoker	10 (25)
Former smoker	29 (73)
Current smoker	1 (3)
Brinkman index, median (IQR)	600 (75, 1028)
Specimen from surgical lung biopsy available, %	100
Median years from diagnosis to nintedanib administration (range)	2.3 (0.1–7.4)
Forced vital capacity	
Median, L (IQR)	2.11 (1.93, 2.66)
Percentage of predicted value, median, % (IQR)	74 (66, 88)
Diffusing capacity of the lung for carbon monoxide	
Median, mmol/min/kPa (IQR)	9.6 (8.1, 12.5)
Percentage of predicted value, median, % (IQR)	56 (49, 65)
Functional progression, n (%)	19 (48)
Radiological progression, n (%)	28 (70)

IQR, interquartile range; Brinkman index calculated as the number of cigarettes smoked per day multiplied by the number of years smoked.

Median age was 66 years and 32 (80%) patients were men; the median time from diagnosis to nintedanib administration was 2.3 years (range: 0.1–7.4 years). In the study cohort, 19 (48%) and 28 (70%) patients exhibiting all-cause death, acute exacerbations, relative FVC decline, or disease progression on chest CT scans in 12 months were defined to have functional or radiological progression, respectively.

### Histopathological characteristics

**Fig 2:**
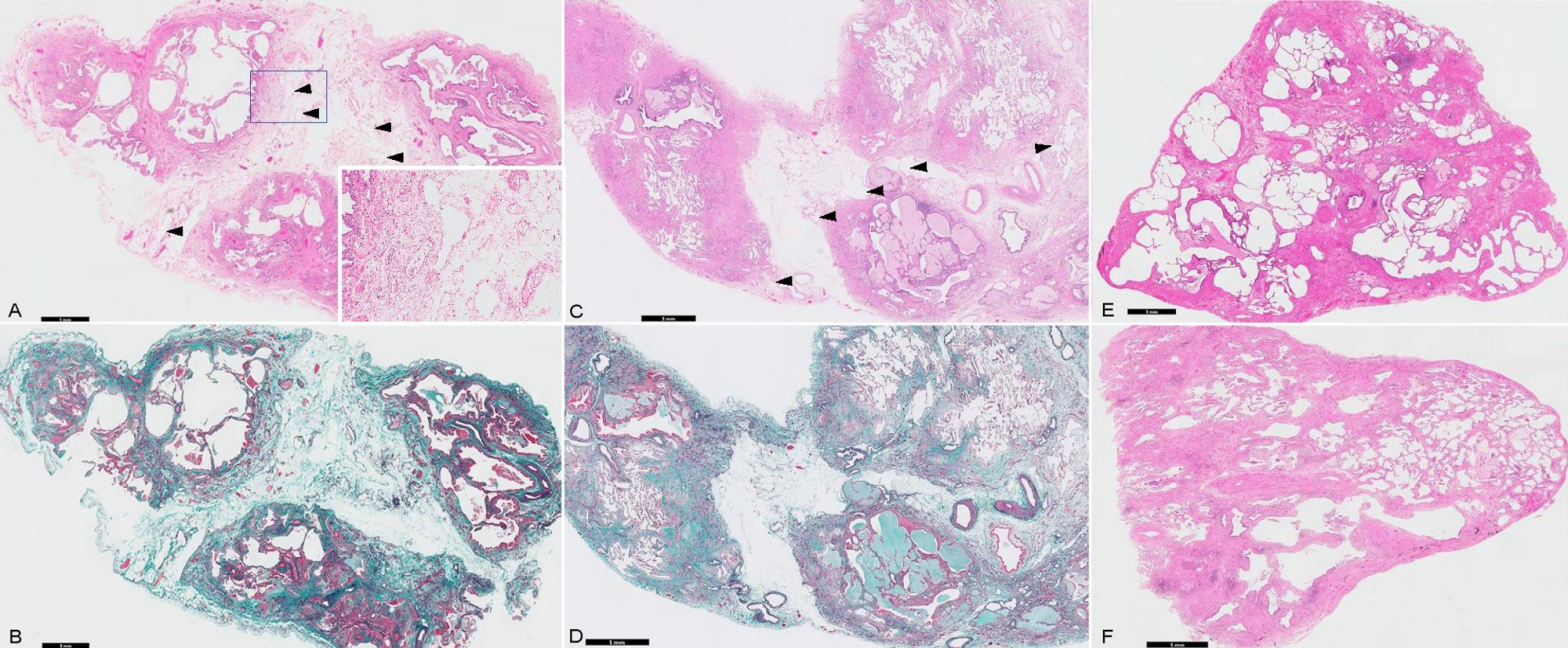
Representative image of edematous changes in the interlobular septum (ECIS) from a surgical lung biopsy sample. (A, B) and (C, D) are two cases of ECIS with remarkable oedema and interlobular septal thickening. The dilated lymph vessels (arrowheads) support the presence of an oedema as a true finding and not an air contamination during the specimen preparation. (E, F) are images of a patient without ECIS. (A, C, E, and F) Haematoxylin and eosin staining. (B, D) Elastica van Gieson staining. The black bars show the magnification.

Histopathologically, most SLB specimens showed DF (83%), DA (75%), FF (50%), and HC (40%), which suggested a specific usual interstitial pneumonia pattern (Table 2) [20]. In comparison with each non-progression group, ECIS (demonstrated in Fig 2) was predominantly observed in patients with functional progression (58%, P = 0.007) and was only observed in patients with radiological progression (50%, P = 0.003); otherwise, only 14% and 0% of patients without functional and radiological progression, respectively, showed ECIS. No histopathological findings were significantly different between the progression and non-progression groups. The inter-rater variability kappa value for all histopathological scores (judged as negative [0–1 points] or positive [2–3 points]), showed moderate agreement (κ = 0.48), including those for ECIS (κ = 0.50). There were no statistical differences in the frequencies of any histological findings between patients with and without ECIS (S1 Table).

**Table 2:** Histological findings of surgical lung biopsy specimens.

Pathological findings	All patients	Functional progression		P-value	Radiological progression		P-value
	n = 40 (%)	Yes	No		Yes	No	
		n = 19	n = 21		n = 28	n = 12	
		(%)	(%)		(%)	(%)	
Dense fibrosis	33 (83)	18 (86)	15 (79)	0.689	22 (79)	11 (92)	0.652
Cellular interstitial pneumonia	2 (5)	2 (11)	0 (0)	0.219	2 (7)	0 (0)	1
Marked organizing pneumonia	2 (5)	0 (0)	2 (10)	0.488	0 (0)	2 (17)	0.085
Fibroblastic focus	20 (50)	10 (53)	10 (48)	1	13 (46)	7 (58)	1
Destroyed architecture	30 (75)	14 (74)	16 (76)	1	19 (68)	11 (92)	0.231
Honeycombing	16 (40)	6 (32)	10 (48)	0.349	10 (36)	6 (50)	0.490
Smooth muscle hyperplasia	12 (30)	6 (32)	6 (29)	1	6 (21)	6 (50)	0.130
Elastosis	7 (18)	3 (16)	4 (19)	1	5 (18)	2 (17)	1
Lymphoid follicles	4 (10)	2 (11)	2 (10)	1	2 (7)	2 (17)	0.570
Cellular/fibrinous pleuritis	0 (0)	0 (0)	0 (0)	NA	0 (0)	0 (0)	NA
Plasma cell infiltration	2 (5)	2 (11)	0 (0)	0.219	2 (7)	0 (0)	1
Constrictive bronchiolitis	2 (5)	2 (11)	0 (0)	0.219	2 (7)	0 (0)	1
Vascular intimal thickness	6 (15)	2 (11)	4 (19)	0.664	4 (14)	2 (17)	1
Loose granuloma	0 (0)	0 (0)	0 (0)	NA	0 (0)	0 (0)	NA
Interstitial giant cell	1 (3)	1 (5)	0 (0)	0.475	1 (4)	0 (0)	1
Peribronchiolar metaplasia	16 (40)	6 (32)	10 (48)	0.349	11 (39)	5 (42)	1
Airway-cantered changes	3 (8)	1 (5)	2 (10)	1	1 (4)	2 (17)	0.209
Epithelial injury	7 (18)	3 (16)	4 (19)	1	6 (21)	1 (8)	0.652
Alveolar exudate	4 (10)	2 (11)	2 (10)	1	3 (11)	1 (8)	1
Edematous changes in the interlobular septum	14 (35)	11 (58)	3 (14)	0.007	14 (50)	0 (0)	0.003

NA, not available.

### Baseline characteristics and outcomes

The baseline characteristics of patients with and without ECIS are presented in Table 3. In the univariate analysis, patients with ECIS were older than patients without it (median 68 vs. 66 years, P = 0.014); moreover, patients with ECIS had a higher female-to-male ratio (43% vs. 11%, P = 0.044), lower %DL_CO_ (median 55% vs. 61%, P = 0.050), lower extent of spared area on chest CT (median 54% vs. 63%, P = 0.050), and marginally significantly greater proportion of honeycomb pattern on chest CT (median 5% vs. 0%, P = 0.051). Multivariate analysis including sex, age, the proportion of honeycomb pattern on chest CT, and initial %DL_CO_ revealed that only age and sex had statistically significant correlation to patients with ECIS.

**Table 3:** Comparison of baseline characteristics in patients with or without ECIS in univariate and multivariate analysis.

Characteristic	With ECIS	Without ECIS	P-value
	n = 14	n = 26	
Age, median, years (IQR)	68 (66, 73)	66 (63, 67)	0.014
Men, No. (%)	8 (57)	23 (89)	0.044
Body mass index (IQR)	24 (23, 28)	23 (21, 26)	0.335
Smoking status, No. (%)			
Never/Former/Current smoker	5 (36)/9 (64)/0 (0)	5 (19)/20 (77)/1 (4)	0.640
Brinkman index, median (IQR)	400 (0, 760)	675 (183, 1133)	0.258
Median years from diagnosis to nintedanib administration (range)	2.5 (0.1 to 7.4)	1.4 (0.1 to 5.6)	0.364
Forced vital capacity, median, L (IQR)	2.20 (2.03, 2.76)	2.08 (1.92, 2.60)	0.379
Percentage of predicted value, median, % (IQR)	74 (66, 83)	73 (66, 80)	0.843
Diffusing capacity of the lung for carbon monoxide			
Median, mmol/min/kPa (IQR)	9.49 (8.39, 11.69)	10.34 (8.84, 13.82)	0.363
Percentage of predicted value, median, % (IQR)	55 (44, 61)	61 (55, 69)	0.050
Distance in the 6-minute walk test, median, m (IQR)	510 (424, 576)	528 (470, 615)	0.278
Serum KL-6, median, U/mL (IQR)	1306 (911, 1851)	1235 (910, 1930)	0.921
Initial CT evaluation, median % (IQR)			
Spared area	54 (44, 58)	63 (50, 72)	0.050
Reticular opacity	24 (17, 28)	16 (13, 24)	0.122
Honeycomb	5 (0, 7)	0 (0, 3)	0.051
Traction bronchial -bronchiolectasis	12 (7, 14)	11 (7, 14)	0.921
Cyst and emphysema	0 (0, 6)	2 (0, 5)	0.509
Ground-glass opacity and air-space consolidation	9 (7, 13)	8 (5, 11)	0.232
Multivariate analysis			
**Characteristic**	**Odds ratio**	**95% confidence interval**	**P-value**
Age	1.31	1.01–1.70	0.040
Sex	12.1	1.17–126.00	0.037
Diffusing capacity of the lung for carbon monoxide	1.02	0.998–1.050	0.071
Honeycomb in initial CT evaluation	0.89	0.66–1.18	0.411

CT, computed tomography; IQR, interquartile range; ECIS, edematous changes in the interlobular septum; KL-6, Krebs von den Lungen-6.

**Fig 3:**
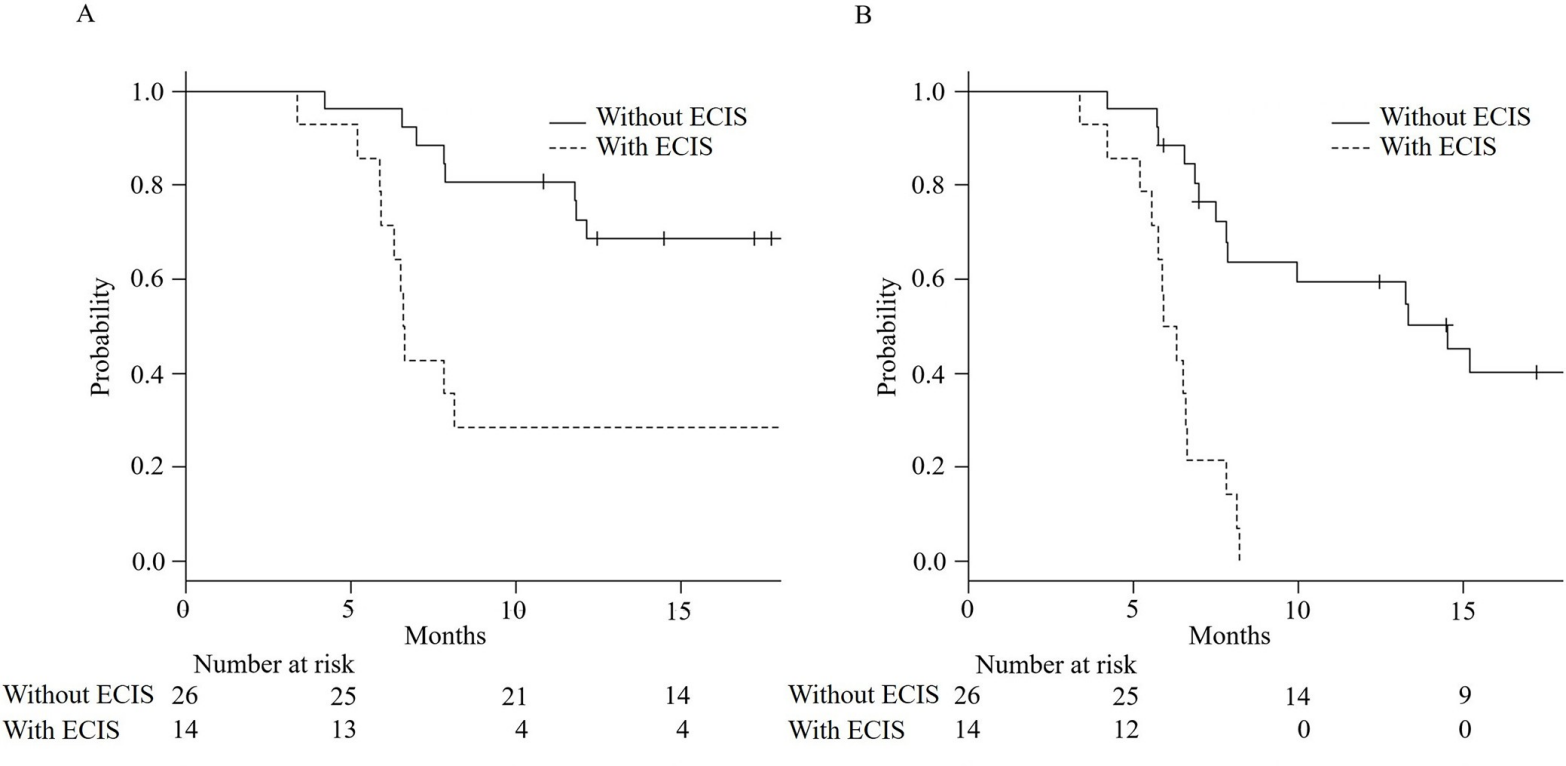
Kaplan–Meier curves for functional (A) and radiological (B) progression-free survival in patients with or without edematous changes in the interlobular septum (ECIS). Both curves (A and B) showed statistical differences (P<0.001 in both).

Functional and radiological PFS were both significantly shorter in patients with ECIS than in those without (6.6 months, 95% confidence interval [CI]: 5.9–25.3, vs. event <50%, 95% CI: 12.1–not available [NA], respectively, P = 0.0009, and 6.1 months, 95% CI: 5.2–6.6, vs. 14.5 months, 95% CI: 7.8–NA, respectively, P<0.0001). Kaplan–Meier curves and comparisons of outcomes for both groups are shown in Fig 3 and Table 4, respectively. There were no significant differences in the numbers of all-cause deaths and acute exacerbations between ECIS and non-ECIS groups. The 12-month progression in PFT and chest CT showed significant differences, and chest CT showed a significant decrease in normal lung area and an increase in the reticular shadow in patients with ECIS. In particular, the median rate of spared area decrease within 12 months was -12% (interquartile range [IQR]: -25%–-5%) of whole-lung volume in patients with ECIS, compared with -3% (IQR: -7%–0%) in patients without ECIS (P = 0.004). The median rate of reticular shadow increase within 12 months was 7% (IQR: 3%–13%) in patients with ECIS, compared with 0% (IQR: 0%–5%) in patients without ECIS (P = 0.041). The inter-rater variability kappa value for the assessment of radiological progression showed high agreement (κ = 0.75).

**Table 4:** Outcome comparison in patients with or without ECIS.

Outcomes	All patients	With ECIS	Without ECIS	P-value
	n = 40	n = 14	n = 26	
All-cause death within 12 months, n (%)	5 (13)	1 (7)	4 (15)	0.640
Acute exacerbation within 12 months, n (%)	5 (13)	3 (21)	2 (8)	0.322
Functional progression, n (%)	19 (48)	11 (79)	8 (31)	0.007
Radiological progression, n (%)	28 (70)	14 (100)	14 (54)	0.003
Change in FVC within 12 months, median, ml (IQR)	-30 (-210, 120)	-180 (-690, 90)	40 (-112, 125)	0.124
Change of spared area on CT within 6 months, median, % (IQR)	-2 (-10, 0)	-7 (-17, -2)	0 (-4, 0)	0.009
Change of spared area on CT within 12 months, median, % (IQR)	-5 (-12, -2)	-12 (-25, -5)	-3. (-7, 0)	0.004
Change of honeycomb on CT within 6 months, median, % (IQR)	0 (0, 0)	0 (0, 0)	0 (0, 0)	0.462
Change of honeycomb on CT within 12 months, median, % (IQR)	0 (0, 0)	0 (0, 3)	0 (0, 0)	0.187
Change of reticular shadow on CT within 6 months, median, % (IQR)	0 (0, 5)	3 (2, 10)	0 (0, 1)	0.001
Change of reticular shadow on CT within 12 months, median, % (IQR)	3 (0, 8)	7 (3, 13)	0 (0, 5)	0.041

CT, computed tomography; FVC, forced vital capacity; IQR, interquartile range; ECIS, edematous changes in the interlobular septum.

## Discussion

This study described the characteristics of patients with IPF undergoing nintedanib treatment, including histopathological findings associated with poor response to therapy. We found that most patients developed functional and radiological progression during the 12-month follow-up period. In patients showing disease progression, ECIS in SLB specimens was significantly more frequently observed; very few patients without functional and radiological progression showed ECIS. In patients with ECIS, PFS was significantly shorter.

To the best of our knowledge, our study is the first to report the histopathological characteristics of patients with IPF who did not respond to nintedanib treatment; we also determined ECIS as an important predictive marker of disease progression. Although the evaluation of some pathological findings is challenging and the level of diagnostic agreement of IPF in previous reports has been very low (κ-values = 0.21 to 0.37) [21–23], the κ-values of interobserver agreement among expert pathologists in the current study remained within the permissive range (κ = 0.48).

According to previous publications, 24–30% of IPF patients with nintedanib developed progression at a rate similar to that of patients without any treatment and showed reduced FVC values during treatment [10]. The current study demonstrated a more frequent progression rate because eligible patients had advanced disease. The surgical lung biopsy itself might cause the increase of acute exacerbation, however the progression-time analysis (Fig 3) showed that there were no patients with acute exacerbation in the first four months after the biopsy. To select study participants, we included patients from the inception of nintedanib administration until January 2017; the median duration of nintedanib administration from the diagnosis of IPF was 2.3 years. Thus, the initial CT evaluation revealed that the median extent of spared area was only 54% in patients with ECIS and 63% in those without ECIS. Even under such circumstances, a notable histological finding of ECIS was predictive of disease progression during nintedanib therapy, especially in the radiological progression group.

To ensure that ECIS was not artifactually induced, we matched tissue processing protocols at both institutions and found that the stepwise specimen processing and reagents used were the same. We also consistently observed dilated lymphatics associated with ECIS (Fig 2), which proved that an edema was a true finding and not an air contamination during the specimen preparation. The background aetiology of ECIS is uncertain. It is one of the histological findings in pulmonary edema; the underlying aetiology of permeability edema has been reported in addition to other mechanisms, such as inhalant toxicity, primary alveolar proteinosis, pulmonary veno-occlusive disease, traumatic lung injury, or acute respiratory distress syndrome. Moreover, it is regarded as a manifestation of lymphovascular disorder in the interstitium or alveolar damage [24–28]. If alveolar capillary damage persists, leakage of the protein-rich fluid from the intravascular space into the interstitium may progress and eventually induce pulmonary fibrosis [29]. Furthermore, ECIS may cause uneven distribution of blood in the lung, which hinders effective drug delivery. In the perspective of respiratory function, the DLco showed slightly high odds ratio for the existence of ECIS in multivariate analysis. This might support the uneven distribution of blood in the lungs. or suggest the coexistence of pulmonary hypertension. Although we could not access full clinical information, the pathological subanalysis did not show the significance of vascular intimal thickness in patients with ECIS. The presence of ECIS may explain the subtypes of IPF not promoted by the major genetic determinants, such as airway mucin gene family, *MUC5B* or *MUC2* [11], but by some factors related to the inflammatory response. Further studies are needed for better pathological understanding of ECIS in patients with IPF and for correlation with other clinical biomarkers, such as bronchoalveolar lavage fluid, right heart catheter, and so on.

This study had some limitations that should be addressed. First, this was a two-centre study based on a relatively limited sample size due to the rare prevalence of the disease [30] and strict participant inclusion and exclusion of only those precisely diagnosed with SLB through multidisciplinary discussion and carefully followed up with multiple PFTs and chest CT scans around 12 months after the nintedanib initiation. Second, this was a retrospective investigation that lacked a control group. Therefore, we cannot exclude the efficacy of nintedanib, even in the progressive disease group. Additionally, switch to or combination with the alternative therapy (e.g., pirfenidone or immunosuppressants) is another important issue in the progressive group that requires further investigation. Third, the observation period in this study was not designed to investigate the difference in survival, as this requires more time; however, it was sufficient for analysing PFS. In addition, it is notable that both functional and radiological progression included the five patients with all-cause death and acute exacerbation for reducing the survivor bias. Fourth, this retrospective cohort without controls could not eliminate the biases and confounders. On the other hand, the fact that no patient without ECIS showed disease progression gave sufficient power to waive multivariate analysis. Last, it has additional impact to specify clinical biomarkers or radiological character for efficacy prediction of nintedanib for the clinical use than for histopathological one. The current results could help elucidate what causes progression in IPF despite anti-fibrotic regimens and make correlations with clinical biomarkers for treatment efficacy prediction.

Conversely, the strengths of the study are obvious. It is the first of its kind to examine in detail the histopathological changes (21 parameters scored semiquantitatively) of IPF patients treated with nintedanib. We found that ECIS, a previously underappreciated histological finding, was correlated with poor prognosis in the study cohort. Hence, certain histopathologic parameters might be of prognostic significance and a careful evaluation of SLB during IPF treatment should be considered.

In conclusion, the current study described characteristics of patients with SLB-confirmed IPF who demonstrated disease progression during nintedanib treatment. ECIS was the key factor predictive of poor treatment efficacy contributing to shortened PFS. Further studies involving larger series of patients are needed to validate the factors predictive of nintedanib efficacy.

## Supporting information

S1 Table Histological findings in patients with and without oedematous changes in the interlobular septum (OCIS). (DOCX)

S1 Dataset Baseline data of the study population (n = 40). (CSV)
