# Supplementary material for: Histologic factors associated with nintedanib efficacy in patients with idiopathic pulmonary fibrosis
Source: PLoS One. 2021 Jan 7;16(1):e0245147. doi: 10.1371/journal.pone.0245147 (PMC7790243; doi:10.1371/journal.pone.0245147)
Supplement: S1 Table — (DOCX) [file pone.0245147.s001.docx]

**S1 Table.** Histological findings in patients with and without oedematous changes in the interlobular septum (OCIS)

| **Pathological findings** | **With OCIS n = 14** | **Without OCIS n = 26** | **P-value** |
| --- | --- | --- | --- |
| Dense fibrosis | 11 (79) | 22 (85) | 0.679 |
| Fibroblastic focus | 6 (43) | 14 (54) | 0.741 |
| Architectural destruction | 11 (79) | 19 (73) | 1.000 |
| Honeycombing | 7 (50) | 9 (35) | 0.500 |
| Smooth muscle hyperplasia | 3 (21) | 9 (35) | 0.484 |
| Elastosis | 0 (0) | 7 (27) | 0.075 |
| Lymphoid follicles | 2 (14) | 2 (8) | 0.602 |
| Vascular intimal thickness | 2 (14) | 4 (15) | 1.000 |
| Peribronchiolar metaplasia | 7 (50) | 9 (35) | 0.500 |
| Airway-centred change | 1 (7) | 2 (8) | 1.000 |
| Epithelial injury | 3 (21) | 4 (15) | 0.679 |
| Alveolar exudate | 3 (21) | 1 (4) | 0.115 |
